# Rapid and Sensitive Determination of Vanillin Based on a Glassy Carbon Electrode Modified with Cu_2_O-Electrochemically Reduced Graphene Oxide Nanocomposite Film

**DOI:** 10.3390/s18092762

**Published:** 2018-08-22

**Authors:** Jingheng Ning, Quanguo He, Xin Luo, Min Wang, Donglin Liu, Jianhui Wang, Jun Liu, Guangli Li

**Affiliations:** 1 School of Chemistry and Biological Engineering, Changsha University of Science & Technology, Changsha 410114, China; ningjingheng@126.com (J.N.); luoxin_gl@126.com (X.L.); 18397412773@163.com (M.W.); dong993@163.com (D.L.); 2 Hunan Key Laboratory of Biomedical Nanomaterials and Devices, School of Life Sciences and Chemistry, Hunan University of Technology, Zhuzhou 412007, China; hequanguo@126.com (Q.H.); junliu@hut.edu.cn (J.L.); guangli010@163.com (G.L.)

**Keywords:** cuprous oxide nanoparticles, electroreduced graphene oxide, vanillin, food analysis

## Abstract

A facile cuprous oxide nanoparticles functionalized electro-reduced graphene oxide modified glassy carbon electrode (denoted as Cu_2_O NPs-ERGO/GCE) was fabricated via a simple physical adsorption and electrochemical reduction approach. Cyclic voltammetry and second-order derivative linear scan voltammetry were used to investigate the electrocatalysis oxidation of vanillin on the Cu_2_O NPs-ERGO/GCE. The compound yielded a well-defined voltammetric response in 0.1 M H_2_SO_4_ at 0.916 V (vs. saturated calomel electrode (SCE)). A linear calibration graph was obtained in the concentration range of 0.1 μM to 10 μM and 10 μM to 100 μM, while the detection limit (S/N = 3) is 10 nM. In addition, the Cu_2_O NPs-ERGO/GCE presented well anti-interference ability, stability, and reproducibility. It was used to detect vanillin sensitively and rapidly in different commercial food products, and the results were in agreement with the values obtained by high performance liquid chromatography.

## 1. Introduction

Vanillin is a phenolic aldehyde and the primary component of the extract of the vanilla bean with the molecular formula C_8_H_8_O_3_ (4-hydroxy-3-methoxybenzaldehyde). Its natural fragrance is attractive and pleasing. It has been widely used as an additive in the food industry. Synthetic vanillin, as an alternative, is now used more often than the natural one as a flavoring agent in foods, beverages, and pharmaceuticals. This compound is applied to contribute to the fragrance of commercial foods such as ice cream, pudding, cookies, beverages, chocolate, and custard [1]. However, studies have shown that vanillin can cause headaches, vomiting, and nausea when ingesting large amounts of this flavor enhancer, and may affect kidney and liver functions [1]. Consequently, the development of vanillin detection methods in foods that are simple and reliable is very important and valuable because of food safety. Until now, several methods for determining vanillin have been described and involve the use of high-performance liquid chromatography [2], gas chromatography [3], thin layer chromatography [4], capillary electrophoresis [5], UV-visible (vis) spectrophotometry [6], and chemiluminometry [7]. However, most of the above methods require highly complex instruments and involve time consuming sample pretreatment processes. As a result of some merits of fast response, high sensitivity, simple operation, and low cost, electrochemical methods have been tried and developed for the detection of vanillin. However, although the molecule is electrooxidizable, there are few studies on the voltammetric determination of vanillin. The main cause is the problem of fouling and regeneration of the electrode surface. An effective way to overcome these obstacles is electrode modification. Some chemically modified electrodes have been reported [1,8,9,10,11,12,13,14,15,16]. The performance of these sensors is heavily dependent on the modified materials. Therefore, the exploration of new materials for the fast and accurate detection of vanillin is greatly demanded.

Nowadays, various nanostructured materials have been developed to improve the performance of electrochemical sensors. Graphene in particular, which is two-dimensional single-layer graphite, has recently received great attention because of its extraordinary performance [17]. Currently, sensors modified with graphene/metal oxides have been widely reported [18,19]. Cuprous oxide (Cu_2_O) is a p-type semiconductor that is considered to be an attractive and promising material because of its relatively narrow band gap (2.0–2.2 eV), low cost, and non-toxic nature. In previous studies, Cu_2_O was successfully used to modify electrode surface to enhance the response signals of H_2_O_2_ [20,21], glucose [21,22,23], dopamine [24,25,26], herbicide paraquat [27], l-tyrosine [28], and NO_2_ [29]. As far as we know, the electrochemical detection of vanillin using a Cu_2_O modified electrode is still missing. However, Cu_2_O nanoparticles are easy to aggregate, which greatly limits its application in electrochemical sensors. How to reduce the aggregation of Cu_2_O nanoparticles has become an urgent problem for researchers. Cu_2_O-reduced graphene oxide nanocomposites have been prepared by Xu et al. [20] using physical adsorption, in situ reduction, and one-pot synthesis. The composites were dispersed in 0.1% Nafion solution and modified on glassy carbon electrode by dropping coating method. The non-enzymatic hydrogen peroxide sensor was constructed. Zhang et al. [24] successfully prepared Cu_2_O/graphene nanocomposites by the solvothermal method. The modified electrode showed good electrocatalytic effect on dopamine. The linear range is 0.1 to 10 µM, and the detection limit is 10 nM. Cu_2_O microparticles and polyvinyl pyrrolidone functionalized graphene nanosheets (micro-Cu_2_O/PVP-GNs) modified glassy carbon-rotating disk electrode (GC-RDE) were prepared by Ye et al. [27]. The sensitive and selective detection of herbicide paraquat was carried out using the excellent catalytic performance of Cu_2_O and GNs. However, the above graphene (GR) based composites require more synthetic steps. Green synthesis of Cu_2_O–GR composite for electrochemical detection of vanillin has not been reported.

In this paper, an efficient, inexpensive, and rapid technique was presented to prepare Cu_2_O NPs–graphene composite via a simple physical adsorption and electrochemical reduction approach. Generally, GR can be synthesized on a large scale by chemical reduction of graphene oxide (GO). In the laborious process, excessive and toxic reducing agents such as hydrazine hydrate and sodium borohydride will contaminate the resulting materials. Compared with the chemical reduction methods, this method is simple, non-toxic, time-saving, and green. Because of its enhanced catalytic activity and high adsorption capacity, the prepared Cu_2_O nanoparticles functionalized electro-reduced graphene oxide modified glassy carbon electrode (NPs-ERGO/GCE) can be used as an effective electrochemical sensor for sensitive determination of vanillin. For further confirmation of the feasibility of practical application, the vanillin contents in some commercial food products were also determined.

## 2. Experiment

### 2.1. Chemical and Solutions

Graphite, hydrazine hydrate solution (80 wt%), cupric sulfate (CuSO_4_·5H_2_O), and vanillin were supplied by Sinopharm Chemical Reagent Co., Ltd., Shanghai, China. H_2_O_2_ solution (30 wt%) and polyvinyl pyrrolidone (PVP) was purchased from Aladdin (Shanghai, China). Vanillin was dissolved in 5% (*v*/*v*) ethanol aqueous solution to prepare a 1.0 × 10^−3^ M standard stock solution, preserved at 4 °C, and protected from daylight when not in use. Working solutions of lower concentrations were prepared by appropriate dilution of the stock solution. The buffer solution and standard solution were prepared by double distilled water. All chemicals are analytical grade and used as received. 

### 2.2. Apparatus

All the electrochemical measurements were measured on a CHI 660E Electrochemical Workstation (Chenhua Instrument Co. Ltd., Shanghai, China) and a Model JP-303E Polarographic Analyzer (Chengdu Instrument Factory, Chengdu, China). A conventional three-electrode system was employed throughout, consisting of a Cu_2_O NPs-ERGO/GCE (3 mm inner diameter) as working electrode, a Pt wire counter electrode, and a saturated calomel reference electrode (SCE).

Solution pH values were measured using a digital pHs-3c Model pH meter (Shanghai Leichi Instrument Factory, Shanghai, China). The morphology of the samples was obtained on a scanning electron microscopy (EVO10, Carl Zeiss Jena GmbH, Jena, Germany). High performance liquid chromatography (HPLC) was conducted on Waters model 510 system (Waters Ltd., Milford, MA, USA) including a 250 mm × 4.6 mm Kromasil 100-5C18 column, using aqueous acetic acid (1%, *v*/*v*)–acetonitrile (85:15, *v*/*v*) as mobile phase with a flow rate of 0.6 mL min^−1^, and equipped with a Waters 2487 dual λ absorbance UV/Vis detector (Waters Ltd., Milford, MA, USA).

### 2.3. Synthesis of Cuprous Oxide 

**Scheme 1:**
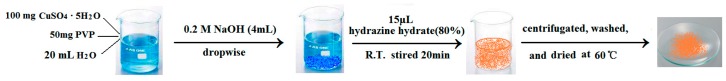
The synthesis of Cuprous oxide (Cu_2_O) nanoparticles. PVP—polyvinyl pyrrolidone. (R.T.: Room Temperature).

Cuprous oxide nanoparticles (Cu_2_O NPs) were prepared according to a previous method [20]. Typically, 100 mg CuSO_4_·5H_2_O and 50 mg PVP was added in 20 mL double distilled water under stirring. Then, 0.2 M NaOH (4 mL) was added dropwise, and blue precipitate came into being. Finally, 15 μL of hydrazine hydrate solution (80 wt%) was added to the mixture and stirred at room temperature for 20 min to form a brick-red suspension. The solid product was separated from the solution by centrifugation, washed with absolute ethanol and water, and dried under vacuum condition of 60 °C. The synthesis route for Cu_2_O NPs can be illustrated as Scheme 1.

### 2.4. Preparation of Cu_2_O–GO Nanocomposite

According to our previous report [25,26,30,31], natural graphite powder is oxidized to graphite oxide using a modified Hummers method. Then, 10 mg graphite oxide was dispersed into 10 mL double distilled water and exfoliated to GO by ultrasonication for 2 h. The unexfoliated graphite oxide was removed by centrifuging at 6000 rpm for 30 min. For preparation of Cu_2_O NPs–GO composite, 1.0 mg Cu_2_O NPs was dispersed into 20 mL GO colloid and then sonicated for about 2 h to give a stable and homogeneous suspension.

### 2.5. Fabrication of Electrochemical Sensor

Before modification, bare GCE was polished to mirror-like by 0.3 μm alumina slurry, and then the GCE was washed with anhydrous alcohol and water successively by ultrasonication for 3 min, respectively, and dried in N_2_ blowing. Then, 5 μL of Cu_2_O NPs–GO was dropped on the freshly prepared pure GCE surface. After drying under ambient condition, the GO film was reduced in a pH 6.0 phosphate buffer at a constant potential of −1.2 V for 120 s, and the Cu_2_O NPs-ERGO/GCE was obtained. GO/GCE, Cu_2_O NPs/GCE, ERGO/GCE, and Cu_2_O NPs-GO/GCE were also prepared with the similar procedures for comparison.

### 2.6. Electrochemical Measurements

Voltammetric measurements were conducted in a 10 mL electrochemical cell containing a desired concentration of vanillin in 0.1 M H_2_SO_4_. Cyclic voltammograms were measured between +0.50 V and +1.15 V at a scan rate of 0.1 V s^–1^. Second-order derivative linear sweep voltammograms were performed from +0.0 V to +1.2 V at a scan rate of 0.1 V s^−1^, after accumulation at 0.0 V for 90 s. When measuring sample solution by second-order derivative linear sweep voltammetry, the background subtraction was employed in order to avoid the distortion of the signal.

## 3. Results and Discussion

### 3.1. Characterization of the GO and Cu_2_O-ERGO Nanocomposites

**Figure 1:**
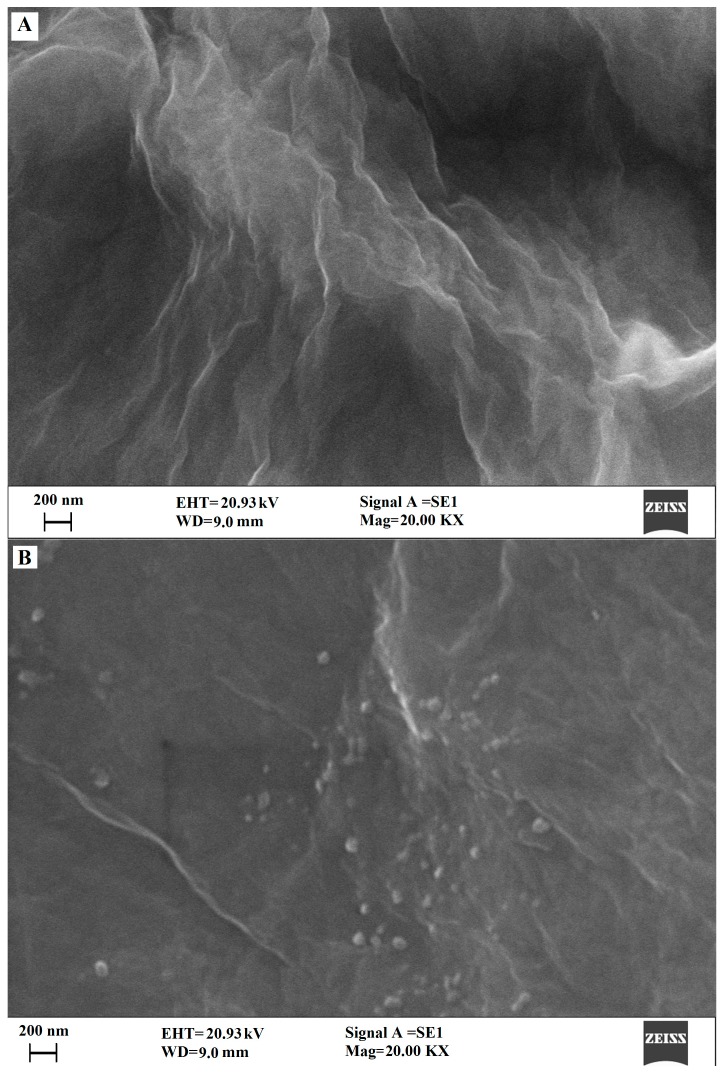
Scanning electron microscopy (SEM) images of (**A**) GO and (**B**) cuprous oxide functionalized electroreduced graphene oxide (Cu_2_O-ERGO) nanocomposite.

The morphologies of GO and Cu_2_O-ERGO nanocomposites were investigated by scanning electron microscopy (SEM). Figure 1A shows the SEM image of GO film. The nanosheets show abundantly wrinkle and crumple-like surface structure, indicating successful preparation of GO. Figure 1B shows the SEM image of Cu_2_O-ERGO nanocomposites produced by the physical adsorption and electrochemical reduction approach. As can be seen, large numbers of Cu_2_O nanoparticles were decorated on a thin film of ERGO, and no particles scattered out of the supports, indicating a strong interaction between graphene support and particles. The average diameter of these particles was about 50–100 nm and most of these particles had a spherical outline. Highly dispersed Cu_2_O on supports with larger surface areas enabled better catalytic activity and sensor sensitivity.

### 3.2. Electrochemical Characterization of Modified Electrodes

**Figure 2:**
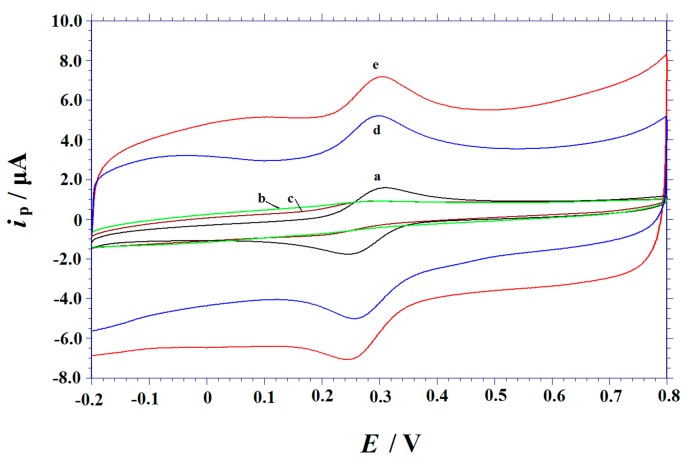
Cyclic voltammograms of 5 mM K_3_[Fe(CN)_6_] in 1.0 M KCl for different electrodes: (**a**) bare glassy carbon electrode (GCE), (**b**) Cu_2_O/GCE, (**c**) GO/GCE, (**d**) ERGO/GCE, and (**e**) Cu_2_O-ERGO/GCE. Scan rate: 0.1 V s^−1^.

The surface status and the barrier of different modified electrodes were monitored by cyclic voltammetry using ferricyanide as redox probe, and the corresponding cyclic voltammograms were shown in Figure 2. There is a pair of Fe(CN)_6_^3−/4−^ reversible redox peaks on bare GCE. The peak potentials were E_pa_ = 0.308 V, E_pc_ = 0.245 V, and the peak currents were i_pa_ = 14.01 μA and i_pc_ = 15.22 μA (curve a). On the Cu_2_O NPs/GCE, the redox peak currents of [Fe(CN)_6_]^3−/4−^ declined greatly. This is because Cu_2_O NPs are easily agglomerated and have poor conductivity, which hinders electron transfer (curve b). A significant decrease in redox peak current was also observed on GO/GCE due to the weak conductivity of GO (curve c). On the ERGO/GCE, the redox peak currents of the [Fe(CN)_6_]^3−/4−^ were improved dramatically with the ΔE_p_ value decreased to 42 mV (curve d). This may be a result of the large specific surface area and high electrical conductivity of ERGO, which increases the concentration of Fe(CN)_6_^3−/4−^ on the electrode surface and promotes the electron transfer rate. When using Cu_2_O NPs-ERGO/GCE, the currents were further increased (curve e), indicating synergistic effect of both Cu_2_O NPs and ERGO, which improves the electron transfer of Fe(CN)_6_^3−/4−^ at the electrode surface.

### 3.3. Electrocatalytic Activity of Cu_2_O NPs-ERGO/GCE Towards Vanillin

**Figure 3:**
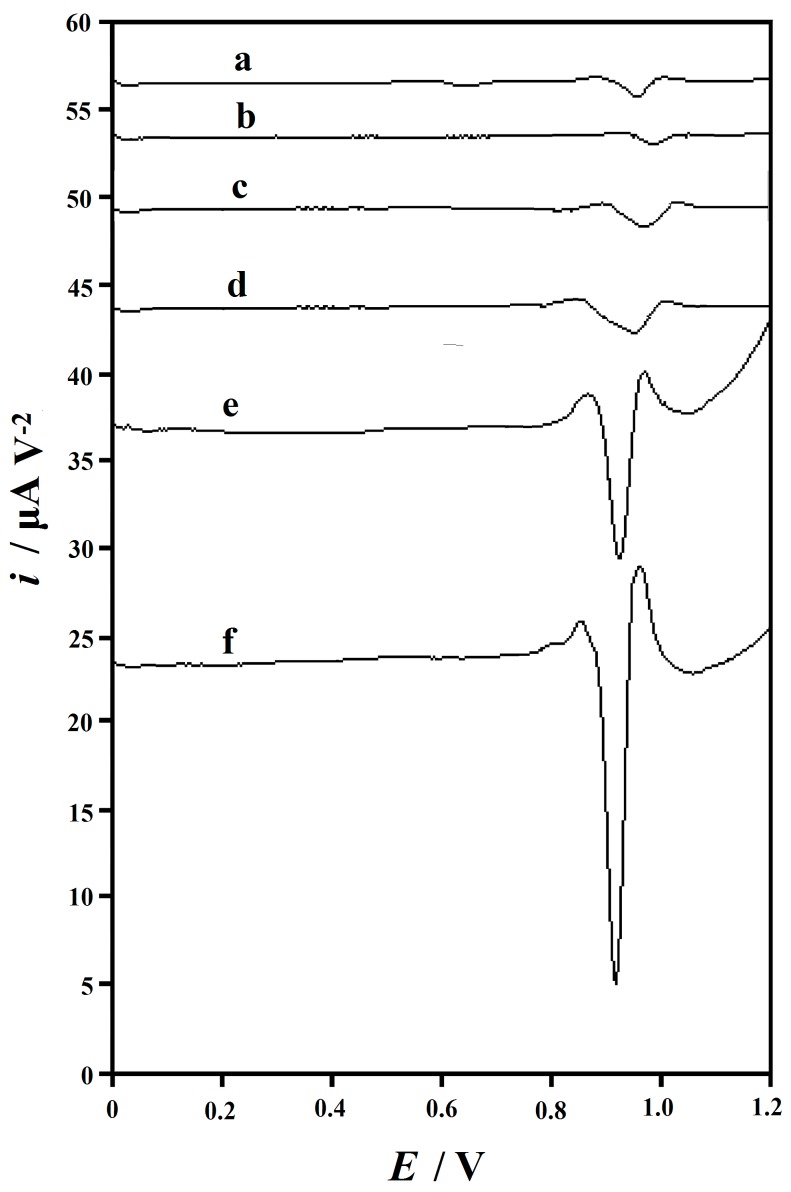
Second-order derivative linear sweep voltammograms of 10 μM vanillin in 0.1 M H_2_SO_4_ solution obtained at different electrodes: (**a**) GCE, (**b**) GO/GCE, (**c**) Cu_2_O nanoparticles (NPs)/GCE, (**d**) Cu_2_O NPs-GO/GCE, (**e**) ERGO/GCE, and (**f**) Cu_2_O NPs-ERGO/GCE. Accumulation potential: 0.0 V, accumulation time: 90 s, scan rate: 0.1 V s^−1^.

Second-order derivative linear sweep voltammetry is a widely used electrochemical method for enhancing sensitivity and specificity in quantitative detection [32,33]; hence, the sensing performance of Cu_2_O NPs-ERGO/GCE towards vanillin was evaluated by second-order derivative linear sweep voltammetry. Figure 3 illustrates the second-order derivative linear sweep voltammetric curves of 10 μM vanillin in 0.1 M H_2_SO_4_ on GCE (curve a), GO/GCE (curve b), Cu_2_O NPs/GCE (curve c), Cu_2_O NPs-GO/GCE (curve d), ERGO/GCE (curve e), and Cu_2_O NPs-ERGO/GCE (curve f) with a scan rate of 0.10 V s^−1^. As shown in Figure 3, after 90 s accumulation at the potential of 0.0 V, a weak oxidation peak (1.102 μA V^−2^) is observed at GCE with a peak potential of about 0.952 V. While the peak current recorded at GO/GCE is much lower (0.5938 μA V^−2^) than that obtained at bare GCE, the oxidation potential shifted positively to 0.986 V, suggesting the retarded electron transfer due to the poor conductivity of GO. The peak current of vanillin increased (i = 1.242 μA V^−2^) at the Cu_2_O NPs/GCE under the same conditions. The catalytic effect of Cu_2_O NPs is not obvious. The main reason may be the easy aggregation of Cu_2_O NPs. Meanwhile, vanillin yielded an anodic peak at 0.946 V on the Cu_2_O NPs-GO/GCE and the according peak current increased to 1.351 μA V^−2^. After reducing at −1.2 V for 120 s, the peak current of vanillin increased greatly (10.62 μA V^−2^) at the ERGO/GCE, a shift towards less positive peak potential (0.920 V) can also be observed. Both the decrease in over-potential and the enhancement in oxidation current may be the result of the unique properties of ERGO such as high specific surface area and excellent conductivity. For Cu_2_O NPs-ERGO/GCE, the peak current increases further (24.43 μA V^−2^) as compared with ERGO/GCE, the peak potential of vanillin shifts more negatively to 0.912 V and its peak shape becomes sharp. These phenomena may be attributed to the synergetic effect between Cu_2_O NPs and ERGO. It confirmed that Cu_2_O NPs-ERGO/GCE has much higher electrocatalytic activity towards the oxidation of vanillin.

### 3.4. Cyclic Voltammetric Behaviors

**Figure 4:**
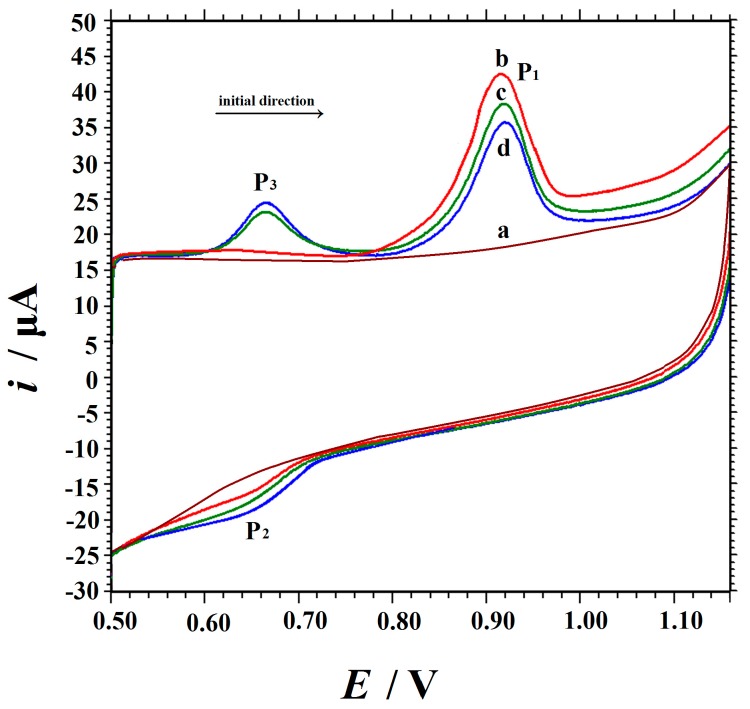
Cyclic voltammetry (CV) curves obtained at Cu_2_O NPs-ERGO/GCE in the absence of vanillin (curve **a**) and in the presence of 10 μM vanillin in 0.1 M H_2_SO_4._ (Curve **b**–**d**: continuous sweep cycles, **b**–**d**: 1st–3rd).

Cyclic voltammetry (CV) is the most widely used technique for acquiring qualitative information about the electrochemical reactions. Figure 4 displays the CV responses on Cu_2_O NPs-ERGO/GCE in the absence and presence of 10 μM vanillin. As shown in Figure 4, no electrochemical response was observed at the Cu_2_O NPs-ERGO/GCE in the blank H_2_SO_4_ solution, indicating that the modified electrode was a non-electrochemically active species over the selected potential range. After the addition of 10 μM vanillin, an oxidation peak (P_1_) was observed on Cu_2_O NPs-ERGO/GCE at 0.920 V attributed to the oxidation of vanillin, and a reduction peak (P_2_) at 0.650 V was also observed in the reverse scan. In addition to P_1_, another oxidation peak (P_3_) at 0.662 V was observed during the subsequent scan, and formed a redox couple with peak P_2_. Simultaneously, compared with that of the first scan, the peak current of P_1_ decreased signally in the subsequent scans, while the redox couple (P_2_/P_3_) increased at the expense of peak P_1_, implying that the product of vanillin by irreversible oxidation remained on or near the surface of the modified electrode and was reduced during the cathodic sweep. The electrochemical behavior of vanillin was consistent with some previous reports [14,15,16].

**Figure 5:**
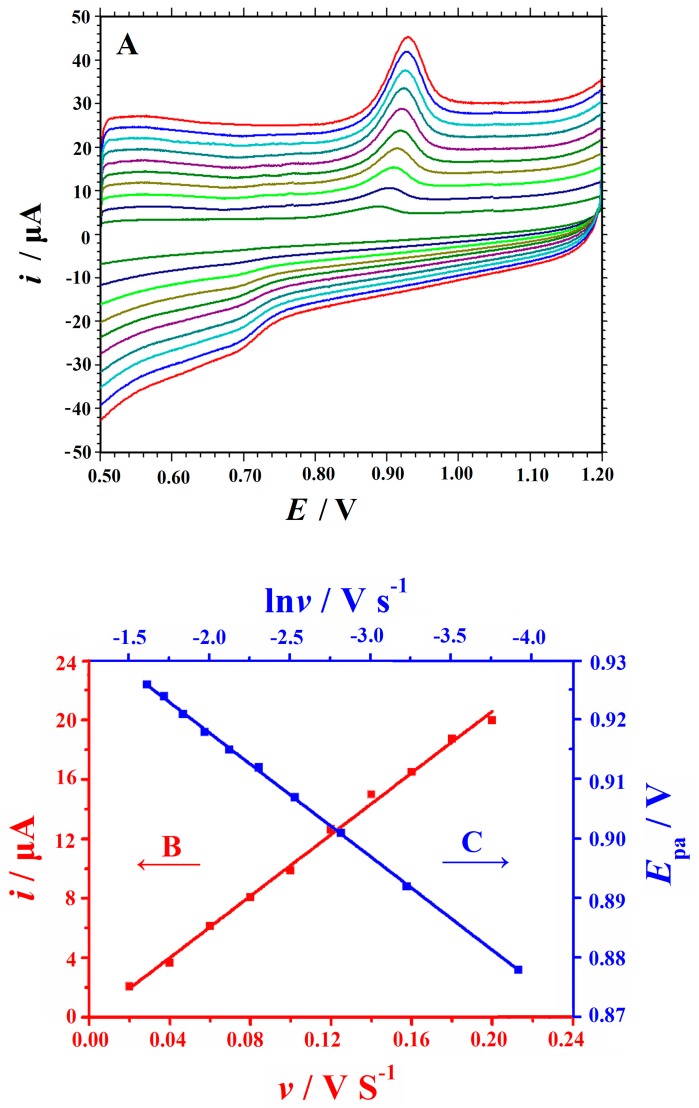
(**A**) CV curves of 10 μM vanillin at Cu_2_O NPs-ERGO/GCE with different scan rates in 0.1 M H_2_SO_4_. From inner to outer: 0.02, 0.04, 0.06, 0.08, 0.10, 0.12, 0.14, 0.16, 0.18, and 0.20 V s^−1^, respectively; the plots for (**B**) the dependence of peak currents (i_pa_) on the scan rates (v) and (**C**) the relationship between peak potentials (E_pa_) and Neperian logarithms of scan rates (ln v).

CV was utilized to study the effect of scan rate on vanillin oxidation at Cu_2_O NPs-ERGO/GCE. The voltammograms were recorded at various scan rates of 0.02 to 0.2 V s^–1^ in the presence of 10 μM vanillin as shown in Figure 5A. The results demonstrated that E_pa_ changes positively with both the i_pa_ increase and the scanning rate increases. This phenomenon is consistent with the characteristics of an irreversible reaction. As shown in Figure 5B, there is a good linear relationship between peak current and scan rate over the range studied. The linear regression equation can be expressed as i_pa_ (A) = 103.66v (V s^−1^) − 0.1335 (R = 0.9983), indicating that the oxidation of vanillin follows the electron transfer process of adsorption control. By plotting logi vs. logv, a straight line with the linear regression equation of log i_pa_ (μA) = 1.0182 log v (V s^−1^) + 2.0261 (R = 0.9984) was obtained. The slope is very close to 1.0, which further confirms the oxidation of vanillin is an adsorption-controlled process. Moreover, in Figure 5C, a plot of E_pa_ versus Neperian logarithm of v (ln v) also presents a linear relationship, and the linear regression equation is E_pa_ (V) = 0.0208 ln v (V s^−^^1^) + 0.9594 (R = 0.9997). According to Laviron’s theory [34], α was assumed as 0.5 for a totally irreversible electrode reaction process [35]. In this work, RT/(αnF) is equal to 0.0208, the calculated number of electrons is about 2.0, which was in accordance with, and exactly the same as, the currently accepted electrooxidation mechanism of vanillin [14,15,16].

### 3.5. Chronocoulometry Study

The electrochemically effective surface areas of the bare GCE and Cu_2_O NPs-ERGO/GCE was investigated and compared by chronocoulometry using 0.1 mM K_3_[Fe(CN)_6_] as model complex. The plots of *Q*–*t* and *Q*–*t*^1/2^ were shown in Figure S1 (Electronic Supplementary Material (ESM)). Equation (1) is given by Anson [36] as follows:

*Q* = 2*nFAcD*^1/2^π^−1/2^*t*^1/2^ + *Q_dl_* + *Q_ads_* (1)

Based on this, the surface area of working electrode *A* could be calculated to be 0.07276 and 0.2362 cm^2^ for GCE and Cu_2_O NPs-ERGO/GCE by the slope of the linear relationship between *Q* and *t*^1/2^ (the diffusion coefficient *D* of K_3_[Fe(CN)_6_] is 7.6 × 10^−6^ cm^2^ s^−1^ [37]). The results showed that the effective surface area of the modified electrode was obviously increased, it would increase the adsorption capacity of vanillin and lead to enhance the current response.

**Figure 6:**
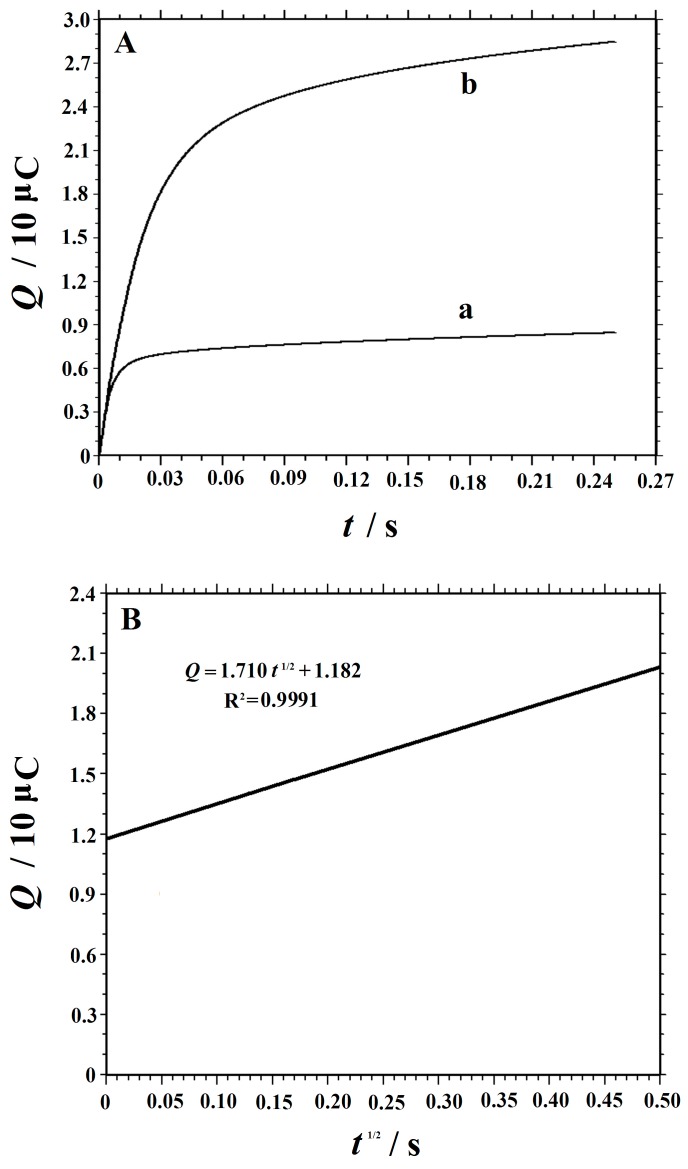
(**A**) Chronocoulometric curves for Cu_2_O-ERGO/GCE in 0.1 M H_2_SO_4_ solution in absence of (a) and in present of (b) 0.1 mM vanillin. (**B**) Plot of *Q*–*t*^1/2^ derived from chronocoulometric curves for Cu_2_O-ERGO/GCE (background subtracted).

The adsorption capacity *Γ*_s_ of vanillin at Cu_2_O-ERGO/GCE can also be calculated by chronocoulometry. As depicted in Figure 6, after point-by-point background subtraction, the charge Q was linear with *t*^1/2^ with a slope of 1.710 × 10^−5^ C s^−1/2^ and *Q_ads_* of 1.182 × 10^−5^ C. The adsorption capacity *Γ*_s_ was calculated to be 2.59 × 10^−10^ mol cm^−2^ according to the equation of *Q_ads_* = *nFAΓ*_s_.

### 3.6. Optimization of Analytical Parameters

#### 3.6.1. Effect of Supporting Electrolytes

The effect of different types of supporting electrolytes on the oxidation peak current of 10 μM vanillin at Cu_2_O-ERGO/GCE was studied. Phosphate buffer (pH 3.0–8.0), HAc–NaAc buffer (pH 4.0–6.0), HAc–NH_4_Ac buffer (pH 4.0–6.0), (CH_2_)_6_N_4_–HCl buffer (pH 4.0–6.0), H_2_SO_4_, HNO_3_, HCl, and KCl (each 0.1 M) were tested. It was found that the highest peak current was observed in H_2_SO_4_. Furthermore, the effect of different acid concentrations on the oxidation of vanillin was also investigated when the concentrations ranges from 0.01 M to 0.5 M. The results showed that the oxidation peak current of vanillin increased gradually with increasing H_2_SO_4_ concentration from 0.01 M to 0.1 M, further beyond the H_2_SO_4_ concentration range, the peak current conversely decreased. Consequently, 0.1 M H_2_SO_4_ was chosen for the subsequent optimizing experiments as the best supporting electrolyte.

#### 3.6.2. Accumulation Potential and Time

**Figure 7:**
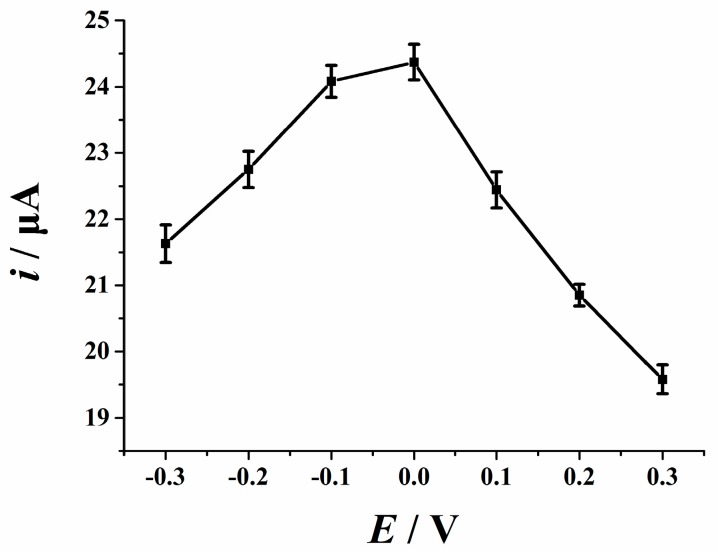
The effects of accumulation potential on the oxidation of 10 µM vanillin in 0.1 M H_2_SO_4_ solution at Cu_2_O NPs-ERGO/GCE. Accumulation time: 90 s, scan rate: 0.1 V s^−1^. Error bars represent SD, *n* = 3.

The relationship between peak current and scan rate described in Section 2.3 showed that the rate-determining step is under adsorption control in the process of vanillin oxidation. Therefore, accumulation can enrich the content of vanillin on the surface of the electrode, thus significantly improving the sensitivity of the determination. The oxidation peak currents of 10 μM vanillin at different accumulative potentials were studied and determined by linear scanning voltammetry. Figure 7 demonstrated that when the accumulative potential changed in the range from 0.30 to 0.0 V, the peak current increased significantly, but decreased under more negative accumulation potentials. Therefore, the accumulative potential of 0.0 V was chosen as the best accumulative potential for the determination of vanillin.

**Figure 8:**
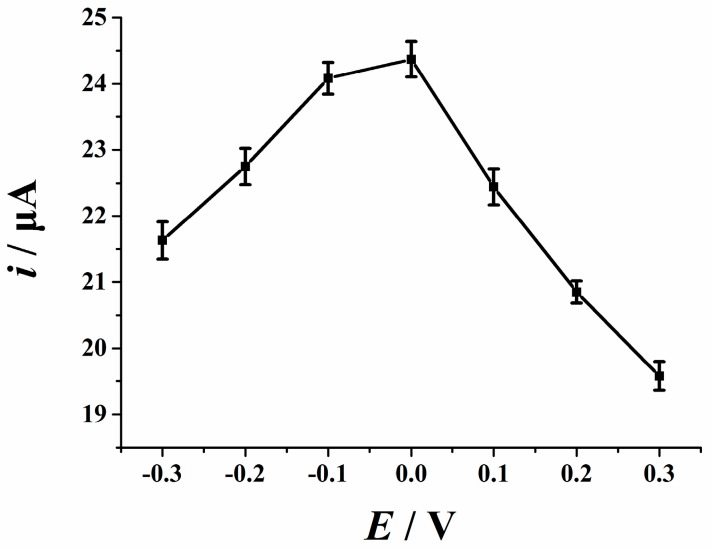
The effects of accumulation time on the oxidation of 10 µM vanillin in 0.1 M H_2_SO_4_ solution at Cu_2_O NPs-ERGO/GCE. Accumulation potential: 0.0 V, scan rate: 0.1 V s^−1^. Error bars represent SD, *n* = 3.

The effect of accumulation time on the peak current of vanillin with a fixed accumulation potential of 0.0 V was investigated. As shown in Figure 8, with the extension of the accumulative time, the oxidation peak current of vanillin gradually increases in the 0−90 s range. However, when the accumulative time exceeds 90 s, the oxidation peak current decreases slightly. This phenomenon might be the result of the supersaturated adsorption of vanillin on the electrode surface, resulting in a certain degree of passivation of Cu_2_O NPs-ERGO/GCE, thus blocking the electron transfer and reducing the response of the modified electrode. Considering two sides of sensitivity and working efficiency, the optimum accumulation time of 90 s was employed.

### 3.7. Analytic Properties

#### 3.7.1. Repeatability, Reproducibility, and Stability

The repeatability, reproducibility, and stability of the developed sensor were evaluated by second-order derivative linear sweep voltammetry. The most attractive feature with the use of Cu_2_O NPs-ERGO/GCE for vanillin detection is the easy renewal of electrode surface for the next use. After each measurement, the electrode surface can be renewed by two simple successive voltammetric sweeps from 0.0 V to 1.2 V in 0.1 M potassium biphthalate solution. Seven measurements of 10 μM vanillin on a single electrode yielded a relative standard deviation (RSD) of 1.6% (Table S1 (ESM)). As for the reproducibility (intersensors), six electrodes were fabricated to detect vanillin in the above solution. The RSD was 4.8% (Table S2 (ESM)), indicating that the Cu_2_O NPs-ERGO/GCE had acceptable reproducibility for analytical applications. The storage stability of the sensor was investigated. The results found that the current response was almost the same by daily use during seven days. After 14 days of storage, only about 7.3% of leakage was found (Table S3 (ESM)).

#### 3.7.2. Interference Studies

To investigate the anti-interference of Cu_2_O NPs-ERGO/GCE, potential interfering substances were added, such as glucose (1.0 mM), fructose (1.0 mM), sucrose (1.0 mM), ascorbic acid (1.0 mM), citric acid (1.0 mM), oxalic acid (1.0 mM), lactic acid (1.0 mM), tartaric acid (1.0 mM), caffeine (1.0 mM), theophylline (1.0 mM), cholesterol (1.0 mM), and uric acid (0.1 mM), and the results are summarized in Table S4 (ESM). It can be found that no significant interference for the detection of 10 μM vanillin was observed from those compounds. However, because of the very similar nature and chemical structure of ethyl vanillin and vanillin, a one-fold amount of ethyl vanillin showed serious interference. The inorganic ions commonly coexisting in real samples were also tested. The results suggest that 100-fold concentration of K^+^, Na^+^, Mg^2+^, Ca^2+^, Zn^2+^, Al^3+^, Cl^−^, SO_4_^2−^, and PO_4_^3−^ has no influence on the detection of 10 μM vanillin. The results showed that the sensor has good selectivity for analysis of vanillin and provides feasibility for real sample analysis.

#### 3.7.3. Calibration and Limit of Detection

**Figure 9:**
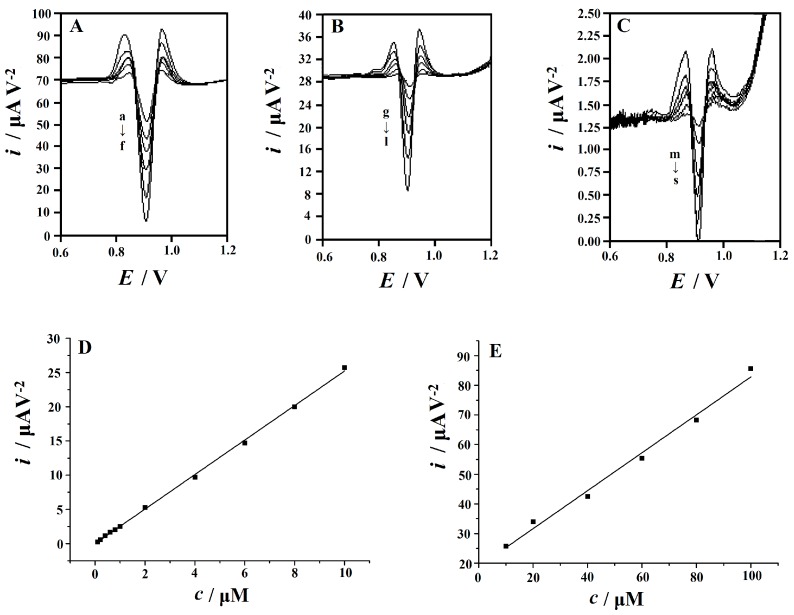
Second-order derivative linear scan voltammograms obtained at Cu_2_O-ERGO/GCE in 0.1 M H_2_SO_4_ solution containing different concentrations of vanillin. (**A**) From a to f: 10, 20, 40, 60, 80, 100 μM; (**B**) From g to l: 1, 2, 4, 6, 8, 10 μM; (**C**) from m to s: 0.1, 0.2, 0.4, 0.6, 0.8, 1.0 μM; (**D**) plot of the oxidation peak currents as a function of vanillin concentrations: 0.1−10 μM (**E**); plot of the oxidation peak currents as a function of vanillin concentrations: 10–100 μM. Accumulation potential: 0.0 V, accumulation time: 90 s, scan rate: 0.1 V s^−1^.

Figure 9 shows the second-order derivative linear sweep voltammetric curves of various concentrations of vanillin on the Cu_2_O NPs-ERGO/GCE. The peak current increased linearly with increment of vanillin concentration in the range of 0.1 μM–10 μM and 10 μM–100 μM. The linear regression equation was expressed as *i* (μA V^−2^) = 2.5222*c* (μM) + 0.0076 and *i* (μA V^−2^) = 0.6394*c* (μM) + 18.889 with correlation coefficient *R* = 0.9995 and 0.9953, respectively. The detection limit was 10 nM (S/N = 3). Table 1 provides a detailed comparison of the properties of different modified electrodes reported for the determination of vanillin. As shown in Table 1, the linear dynamic range reported in our work is wider than most of the previous reports [1,8,9,10,11,14,15]. Although the limit of detection in this work is higher than the Ag-Pd bimetallic nanoparticles-decorated graphene oxide modified glassy carbon electrode (Ag-Pd/GO/GCE) [13] and manganese dioxide nanoflowers-graphene oxide modified glassy carbon electrode [14], this method has made significant progress in simplifying electrode preparation, saving time and reducing costs.

#### 3.7.4. Analytical Applications

In order to evaluate the potential application of this newly-developed method in actual sample analysis, the content of vanillin was determined in different foods such as biscuit, chocolate, candy, and custard. These samples were purchased from a local market and pretreated according to our previous report [16]. Each pretreated sample solution was transferred to a voltammetric cell and analyzed in the day of preparation according to the above-described procedure. Table 2 presented the determination results for four parallel measurements, which were measured by the standard addition method. The recoveries of vanillin standard added into the samples were in the range of 96.3–101.2%, indicating that this method has good accuracy. For comparison, high performance liquid chromatography (HPLC) was used as a reference method to determine the content of vanillin in these samples. As displayed in Table 2, the results obtained by this method are the same as those obtained by HPLC method. The results proved that the modified electrode has good analytical performance and can be a feasible sensor for detecting vanillin in commercial food samples.

## 4. Conclusions

In this work, a homogeneous dispersion was obtained by dispersing cuprous oxide nanoparticles into graphene oxide solution, and a uniform cast film of Cu_2_O NPs-ERGO was achieved via electroreduction method. Compared with bare GCE and ERGO/GCE, the oxidation peak current of vanillin was significantly increased and the oxidation overpotential was decreased at the Cu_2_O NPs-ERGO/GCE. The electrochemical behavior of vanillin on the modified electrode is an absorption-controlled process, involving two electrons with two proton transfers. The peak current was linearly related to the concentration of vanillin, ranging from 0.1 μM–10 μM and 10 μM–100 μM, and the detection limit was 10 nM (S/N = 3). The prepared Cu_2_O NPs-ERGO/GCE not only had strong catalytic activity for vanillin oxidation, but also provided significant quantitatively reproducible analytical performance. This newly developed method has some obvious advantages such as simplicity, quick response, high sensitivity, and low cost. 

## Figures and Tables

**Table 1:** Comparison with other electrochemical methods for the determination of vanillin.

Electrochemical Sensors	Technique	Supporting Electrolyte	Linear Range/μM	Correlation Coefficient	Sensitivity (μA/μM)	Detection Limit/μM	References
^a^ BDD electrode	^i^ SWV	0.1 M PBS, pH 2.5	3.3–98	0.999	0.5984 (μA mL μg^−1^)	0.16	[1]
^b^ AuPd-graphene/GCE	^j^ DPV	0.1 M PBS, pH 7.0	0.1−7 and 10−40	0.996 and 0.999	0.113 and 0.0125	0.02	[2]
^c^ CDA/Au–AgNPs/GCE	Amperometry	0.1 M PBS, pH 2.0	0.2−50	-	-	0.04	[9]
disposable screen-printed electrode	SWV	0.1 M PBS, pH 7.4	5−400	0.9994	0.0265	0.4	[10]
graphene/GCE	DPV	Na_2_HPO_4_− C_6_H_8_O_7_ buffer, pH 5.0	0.6−48	0.9996	0.1523	0.056	[11]
^d^ MWCNTs-TAPcCo/GCE	SWV	0.1 M PBS, pH 7.2	4.2−5000	0.9993	0.04725	0.44	[12]
^e^ Ag-Pd/GO/GCE	DPV	0.1 M PBS, pH 6.0	0.02–45	0.9933	0.5733	0.005	[13]
^f^ Ag NPs/GN/GCE	SWV	0.1 M PBS, pH 6.98	2−100	0.998	0.959	0.332	[14]
^g^ MFG/GCE	DPV	B–R buffer (pH 1.81)	0.03−8	0.995	0.5733	0.0015	[15]
^h^ GR-PVP/ABPE	Derivative Voltammetry	0.1 M H_3_PO_4_	0.02−2.0, 2.0−40, 40−100	0.9985, 0.9959, 0.9985	0.1714; 0.0805; 0.0269	0.01	[16]
Cu_2_O-ERGO/GCE	Derivative Voltammetry	0.1 M H_2_SO_4_	0.1−10; 10−100	0.9995 0.9953	2.5222; 0.6394	0.01	This work

^a^ Boron-doped diamond electrode; ^b^ Au-Pd nanoparticles−graphene composite modified glassy carbon electrode; ^c^ cellulose diacetate/Au–Ag alloy nanoparticlesmodified glassy carbon electrode; ^d^ multi-walled carbon nanotubes chemically modified by 2,9,16,23-tetraaminophthalocyaninatocobalt modified glassy carbon electrode; ^e^ Ag-Pd bimetallic nanoparticles–decoratedgraphene oxide modified glassy carbon electrode; ^f^ novel silver nanoplates/graphene compositemodified glassy carbon electrode; ^g^ manganese dioxidenanoflowers–graphene oxidemodified glassy carbon electrode; ^h^ graphene–polyvinylpyrrolidone composite filmmodified glassy carbon electrode; ^i^ square-wave voltammetry; ^j^ differential pulse voltammetry.

**Table 2:** Determination of vanillin in food samples (*n* = 4). HPLC—high performance liquid chromatography.

Sample ^a^	Found ^b^/μM	Added/μM	Total Found ^b^/μM	Recovery/%	Content ^b^/μg g^−1^	Content Determined by HPLC ^b^/μg g^−1^
Biscuit	0.84(±0.04)	0.80	1.61(±0.03)	96.3	25.56(±1.10)	25.34 (±1.24)
Chocolate	6.18(±0.24)	6.00	12.25(±0.11)	101.2	188.04(±7.17)	187.81 (±6.86)
Candy	1.27(±0.06)	1.00	2.25(±0.17)	98.0	38.64(±1.72)	38.95(±1.54)
custard	5.32(±0.22)	5.00	10.16(±0.47)	96.8	161.87(±6.69)	161.52 (±5.70)

^a^ All samples were collected from local supermarkets. ^b^ Average ± confidence interval, the confidence level is 95%.

## Supplementary Materials

The following are available online at http://www.mdpi.com/1424-8220/18/9/2762/s1.
