# Supplementary material for: Rapid and Sensitive Determination of Vanillin Based on a Glassy Carbon Electrode Modified with Cu2O-Electrochemically Reduced Graphene Oxide Nanocomposite Film
Source: Sensors (Basel). 2018 Aug 22;18(9):2762. doi: 10.3390/s18092762 (PMC6164793; doi:10.3390/s18092762)
Supplement: Supplementary file 1 [file sensors-18-02762-s001.pdf]

Electronic Supplementary Material

Rapid and sensitive determination of vanillin based on a glassy carbon electrode modified with Cu_2O-electrochemically reduced graphene nanocomposite film

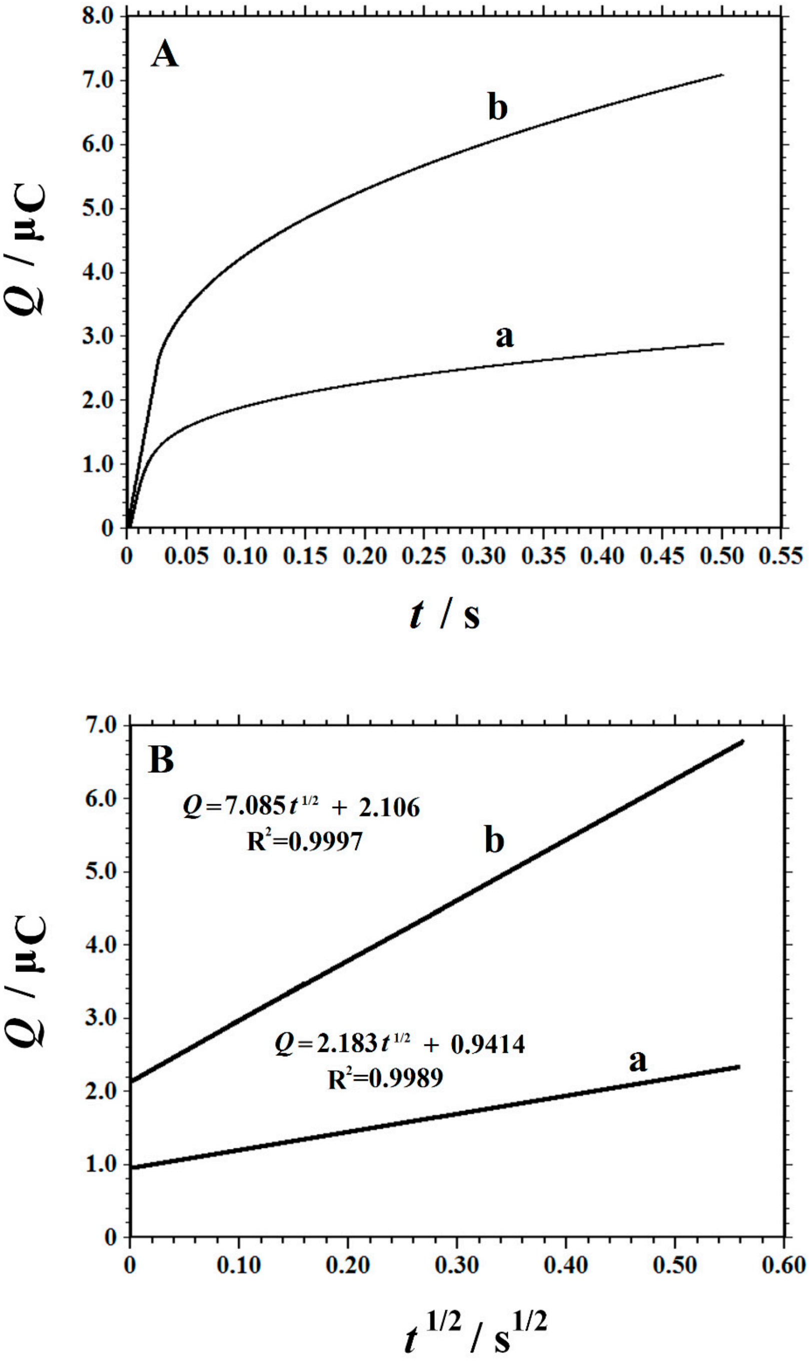

Figure S1. (A) Chronocoulometric curves obtained at GCE (a) and Cu_2O-ERGO/GCE (b) in the presence of 0.1 mM $K_3[Fe(CN)_6]$ containing 1.0 M KCl; (B) Plots of Q-$t^{1/2}$ derived from chronocoulometric curves for GCE (a) and Cu_2O-ERGO/GCE (b).

Table S1. Repeatability of Cu_2O-ERGO/GCE

Determination times	1	2	3	4	5	6	7
Peak current / µA	24.76	25.27	25.31	24.57	25.42	24.86	25.68
Relative standard deviation (RSD)				1.6%			

Table S2. Reproducibility of Cu_2O-ERGO/GCE

Electrode number	1	2	3	4	5	6
Peak current / µA	23.88	25.71	24.93	25.17	22.38	24.35
Relative standard deviation (RSD)			4.8%			

Table S2. The storage stability of Cu_2O-ERGO/GCE

Days	1	2	3	4	5	6	7
Peak current / µA	25.36	25.24	25.17	25.08	25.19	25.04	24.97
Days	8	9	10	11	12	13	14
Peak current / µA	24.84	24.52	24.26	24.05	23.87	23.61	23.50

Table S4. Influence of coexisting substances on the determination of 10μM vanillin

Coexisting substance	Concentratio n / mM	Change of peak current / %	Coexisting substance	Concentration / mM	Change of peak current / %
glucose	1.0	2.87	ethyl vanillin	0.01	17.85
fructose	1.0	-1.16	K^+	1.0	1.89
sucrose	1.0	1.03	Na^+	1.0	1.76
ascorbic acid	1.0	0.62	Mg^{2+}	1.0	-1.23
citric acid	1.0	1.55	Ca^{2+}	1.0	1.42
oxalic acid	1.0	3.17	Zn^{2+}	1.0	1.89
lactic acid	1.0	-2.84	Al^{3+}	1.0	2.35
caffeine	1.0	1.69	Cl^-	1.0	1.94
theophylline	1.0	2.67	SO_4^{2-}	1.0	2.31
cholesterol	1.0	-1.04	PO_4^{3-}	1.0	3.04
uric acid	0.1	3.56	-	-	-
